# The Law Case of Drs. Bower and Keates

**Published:** 1884-07-20

**Authors:** 


### THE LAW CASE OF DRS. BOWER AND KEATES.

The legal case of Drs. Bower and Keates, which has excited so much interest throughout the medical profession in England, has come to a happy termination by a verdict in favor of the defendants. The facts of the case are these: Drs. Bower and Keates, in professional attendance upon a case of diphtheria in a child, found it necessary to perform tracheotomy and to introduce a tube. The father of the child brought action on the ground of alleged negligence of the two medical attendants, who told him to suck the tracheotomy tube when it was first inserted, without warning him of the danger of infection. The case was tried by the Lord Chief Justice, who emphatically expressed the opinion that the medical men were quite justified in asking the father to suck the tube. The jury also expressed this opinion and returned a verdict for the defendants.

The case is of real interest, as showing the sentiments of the medical profession of England in reference to an appeal to defray the heavy legal expenses to which these physicians were subjected. An appeal was made for funds, and the response was so prompt and liberal that, within three weeks, the honorary secretaries in charge of the fund were forced to cry, "Hold! enough." The case is also memorable on account of its legal importance and its results to legal medicine.—*Maryland Medical Journal.*

